# Regulation of Pancreatic β Cell Mass by Cross-Interaction between CCAAT Enhancer Binding Protein β Induced by Endoplasmic Reticulum Stress and AMP-Activated Protein Kinase Activity

**DOI:** 10.1371/journal.pone.0130757

**Published:** 2015-06-19

**Authors:** Tomokazu Matsuda, Hiroaki Takahashi, Yusuke Mieda, Shinobu Shimizu, Takeshi Kawamoto, Yuki Matsuura, Tomoko Takai, Emi Suzuki, Ayumi Kanno, Maki Koyanagi-Kimura, Shun-ichiro Asahara, Alberto Bartolome, Norihide Yokoi, Hiroshi Inoue, Wataru Ogawa, Susumu Seino, Yoshiaki Kido

**Affiliations:** 1 Division of Diabetes and Endocrinology, Department of Internal Medicine, Kobe University Graduate School of Medicine, Kobe, Japan; 2 Division of Medical Chemistry, Department of Biophysics, Kobe University Graduate School of Health Sciences, Kobe, Japan; 3 Naomi Berrie Diabetes Center, Department of Medicine, Columbia University, New York, United States of America; 4 Division of Molecular and Metabolic Medicine, Department of Physiology and Cell Biology, Kobe University Graduate School of Medicine, Kobe, Japan; 5 Department of Physiology and Metabolism, Brain/Liver Interface Medicine Research Center, Kanazawa University, Kanazawa, Japan; Institut d'Investigacions Biomèdiques August Pi i Sunyer, SPAIN

## Abstract

During the development of type 2 diabetes, endoplasmic reticulum (ER) stress leads to not only insulin resistance but also to pancreatic beta cell failure. Conversely, cell function under various stressed conditions can be restored by reducing ER stress by activating AMP-activated protein kinase (AMPK). However, the details of this mechanism are still obscure. Therefore, the current study aims to elucidate the role of AMPK activity during ER stress-associated pancreatic beta cell failure. MIN6 cells were loaded with 5-amino-1-β-D-ribofuranosyl-imidazole-4-carboxamide (AICAR) and metformin to assess the relationship between AMPK activity and CCAAT enhancer binding protein β (C/EBPβ) expression levels. The effect of C/EBPβ phosphorylation on expression levels was also investigated. Vildagliptin and metformin were administered to pancreatic beta cell-specific C/EBPβ transgenic mice to investigate the relationship between C/EBPβ expression levels and AMPK activity in the pancreatic islets. When pancreatic beta cells are exposed to ER stress, the accumulation of the transcription factor C/EBPβ lowers the AMP/ATP ratio, thereby decreasing AMPK activity. In an opposite manner, incubation of MIN6 cells with AICAR or metformin activated AMPK, which suppressed C/EBPβ expression. In addition, administration of the dipeptidyl peptidase-4 inhibitor vildagliptin and metformin to pancreatic beta cell-specific C/EBPβ transgenic mice decreased C/EBPβ expression levels and enhanced pancreatic beta cell mass in proportion to the recovery of AMPK activity. Enhanced C/EBPβ expression and decreased AMPK activity act synergistically to induce ER stress-associated pancreatic beta cell failure.

## Introduction

Pancreatic beta cell mass can be affected negatively by events that disrupt cellular homeostasis, such as oxidative stress or autophagic dysfunction. In particular, endoplasmic reticulum (ER) stress due to obesity and systemic insulin resistance is one important pathogenic factor that might lead to pancreatic beta cell failure [1,2]. However, the details of ER stress-related beta cell failure and onset of diabetes are obscure.

The CCAAT enhancer-binding protein (C/EBP) family of basic leucine-zipper transcription factors includes C/EBPα, -β, -γ, -δ, and -ε, as well as C/EBP homology protein (CHOP) [3]. C/EBPβ performs diverse functions, including the regulation of genes that contribute to the acute phase response, glucose metabolism, and tissue differentiation, including adipogenesis and hematopoiesis [4]. We have shown that the transcription factor C/EBPβ, which is expressed at low levels under normal circumstances, is highly induced by ER stress in pancreatic beta cells [5]. The accumulation of C/EBPβ weakens these cells against ER stress and reduces pancreatic beta cell mass by inhibiting induction of the molecular chaperone 78-kDa glucose-regulated protein (GRP78), which is the major ER chaperone in all eukaryotes that enables the essential process of productive folding in the ER [6–9]. More recently, it has been reported that accumulation of C/EBPβ is also observed in the pancreatic beta cells of type 2 diabetes patients but is not found in patients with normal glucose tolerance [10]. Elucidation of the mechanisms that control C/EBPβ expression is therefore important to discovering novel therapeutic targets for ameliorating pancreatic beta cell failure.

AMP-activated protein kinase (AMPK) is activated by a decrease in cellular energy (an elevation of the AMP/ATP ratio) and restores ATP levels by deactivating biosynthetic pathways and activating catabolism. AMPK activation reportedly reduces ER stress and rescues beta cell function in a cellular model of glucotoxicity [11]. It is noteworthy that C/EBPβ expression is highly sensitive to AMPK activation in the liver [12]. These reports led us to hypothesize that differential interaction between AMPK and C/EBPβ may be key to determining the fate of pancreatic beta cells exposed to ER stress.

In this study, we demonstrated that during the onset of type 2 diabetes, pancreatic beta cells exhibit enhanced C/EBPβ expression along with decreased AMPK activity, which forms a vicious cycle that reduces pancreatic beta cell mass.

## Materials and Methods

### Mice

Pancreatic beta cell-specific C/EBPβ transgenic (TG) mice with a C57BL/6J background were generated and maintained as described previously [5,13,14]. Male wild-type and C/EBPβ TG mice were grouped and housed with access to either regular water or water continuously supplemented with metformin (LSG Corporation, Tokyo, Japan) and/or 0.6 mg/mL vildagliptin (a gift from the Novartis Institutes for BioMedical Research, Cambridge, MA, USA) from 4 to 12 weeks of age. Mice were sacrificed after the study by cervical dislocation. This study was approved by the Animal Ethics Committee of Kobe University Graduate School of Medicine (approval number P130508).

### Cell culture and transfection of siRNA

MIN-6 cells were maintained in Dulbecco’s modified Eagle's medium supplemented with 15% heat-inactivated fetal bovine serum and 1% penicillin/streptomycin. For overexpression of C/EBPβ, MIN-6 cells were transfected with expression plasmid carrying the full C/EBPβ by using Lipofectamine 3000 (Invitrogen) transfection reagent. For knockdown of AMPK, MIN-6 cells were re-plated in 12-well plates (60-mm dishes) at 24 h before transfection and transfected with siRNA for AMPKα1 and α2 (SMARTpool; Dharmacon, Lafayette, CO) or scramble controls (Non-Targeting siRNA#2; Dharmacon) with DharmaFECT2 transfection reagent (Dharmacon). After 48 h of further incubation for 48 h for protein, cells were harvested for evaluation of C/EBPβ expression.

### Oral glucose tolerance test

Mice were deprived of food for 16 h prior to the oral administration of glucose (1.5 mg/g body weight). Blood was collected immediately before and at 15, 30, 45, 60, and 120 min after glucose administration.

### Islet isolation

Pancreatic islets were isolated by collagenase digestion and hand-picked under a stereoscope as previously described [14,15].

### Immunostaining and morphometric analysis

The pancreas was immersed in Bouin’s solution, embedded in paraffin, and sectioned at a thickness of 4–5 μm. Sections were stained with antibodies against insulin and glucagon (Dako Japan, Kyoto, Japan). Immune complexes were detected using secondary antibodies conjugated to either Cy3 or FITC (Jackson ImmunoResearch Laboratories, West Grove, PA, USA). Quantitation of beta cell mass was performed as described previously [13].

### Immunoblot analysis

Lysates of the isolated islets and C/EBPβ-overexpressing MIN6 cells were prepared as described previously [5,14] and probed with antibodies against phosphorylated (p)-C/EBPβ (Thr235: equivalent to Thr188 of the mouse sequence; Cell Signaling, Danvers, MA, USA), C/EBPβ, CHOP (Santa Cruz Biotechnology, Inc., Santa Cruz, CA, USA), p-AMPK (Thr172), AMPK, c-Myc, acetyl-CoA carboxylase (ACC), p-ACC, eukaryotic translation initiation factor 2α (eIF2α), p-eIF2α, p-c-jun (Cell Signaling Technology, Danvers, MA, USA) anti-human influenza hemagglutinin (Roche Applied Science, Mannheim, Germany), and β-actin (Sigma-Aldrich, St. Louis, MO, USA). Quantitative analysis of blots was performed with Multi Gauge Version 3.0 software (Fujifilm, Tokyo, Japan), and protein levels were normalized to the β-actin levels.

### Plasmid construction

C/EBPβ mutant vectors (T188A, E) were prepared by cloning wild-type mouse C/EBPβ (35 kDa isoform) cDNA into the pBABE vector. The mutants were generated using a PrimeSTAR Mutagenesis Basal Kit (Takara Bio, Inc., Otsu, Japan). pcDNA3 vectors containing wild-type or dominant negative (DN) (K45R) AMPK were obtained from Addgene (Plasmids #15991 and #15992; Cambridge, MA, USA). The pcDNA3 vector containing the constitutively active (CA) (T172D) AMPK sequence was a gift of David Carling (Imperial College, London, UK). All cDNA sequences were further cloned into the pBABE vector.

### Metabolomic analysis

For metabolite extraction, 450 μl MilliQ water containing internal standards (methionine sulfone and camphor-10-sulfonic acid), 500 l CHCl_3,_ and 250 μl methanol were added to 30 mg tissue or 300 pancreatic islets, and the mixture was homogenized by a bead mill homogenizer. After centrifugation, the supernatant was collected, filtered using a 5-kDa cutoff filter, and freeze-dried. The hydrophilic metabolites were subjected to capillary electrophoresis-mass spectrometry on an Agilent capillary electrophoresis coupled to time-of-flight mass spectrometry (7100 CE and 6224 TOF LC/MS). Metabolite concentrations were calculated as the ratio between each metabolite in the sample and the standard compounds.

### Statistical analysis

Results are expressed as the mean ± standard error. Statistical significance was assessed with ANOVA and, when appropriate, Student’s *t* test. A *P* value of < 0.05 was considered statistically significant.

## Results

### Enhanced C/EBPβ expression caused by ER stress decreases AMPK activity

**Fig 1:**
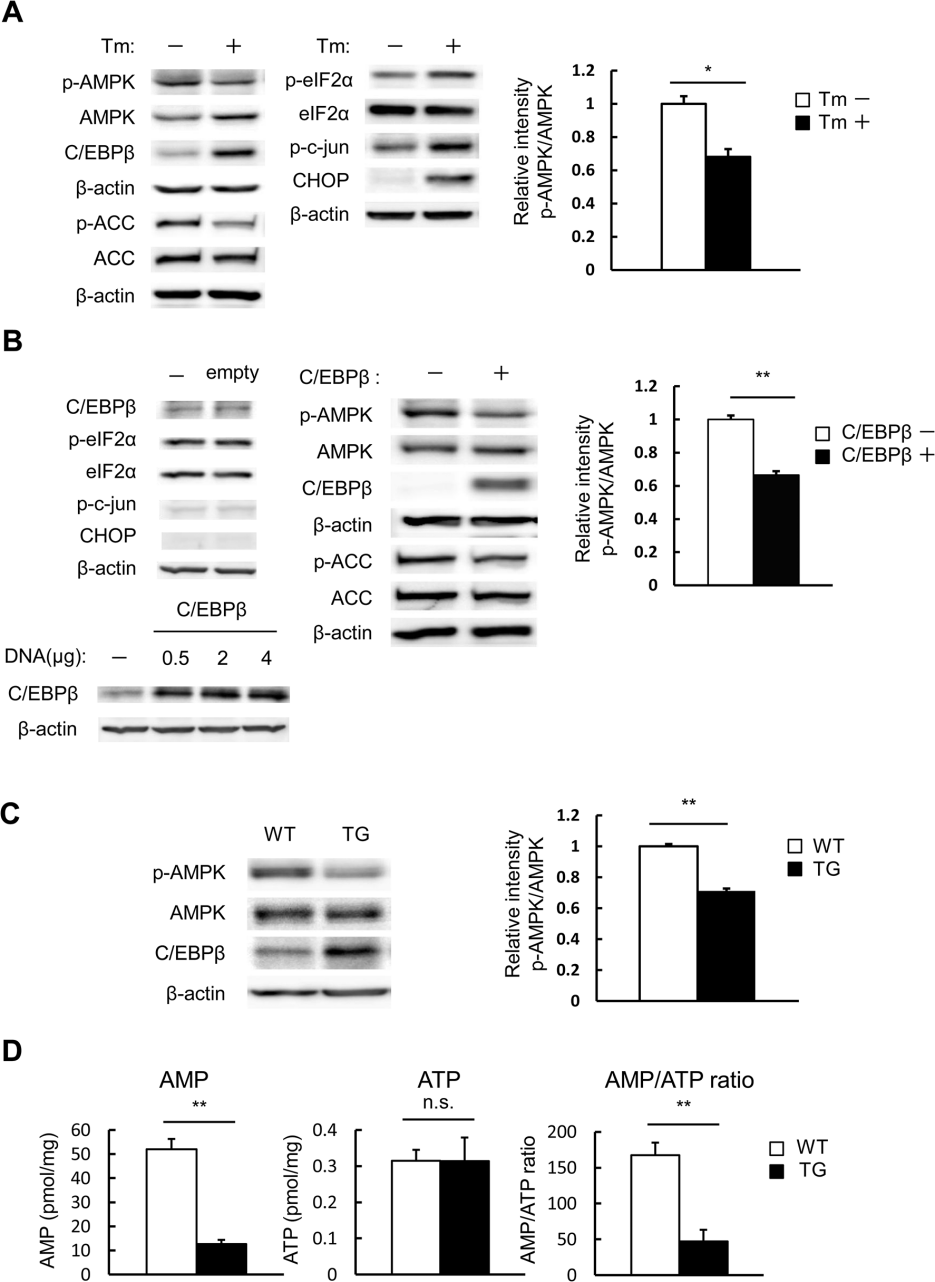
Accumulation of C/EBPβ due to ER stress lowers AMPK activity. A: MIN6 cells were treated with 1 μg/mL tunicamycin (Tm) for 24 h and analyzed with the indicated antibodies (left). Quantitation of the AMPK phosphorylation level is normalized to total AMPK (right). B: MIN6 cells were transfected with C/EBPβ expression vectors or empty vector. The cells were analyzed with the indicated antibodies (left and middle). Quantitation of the AMPK phosphorylation level is normalized to total AMPK (right). C: Isolated islets from wild-type (WT) and TG mice at 12 weeks of age were analyzed with the indicated antibodies (left). Quantitation of the AMPK phosphorylation level is normalized to total AMPK (right). D: Isolated islets from WT and TG mice at 12 weeks of age were analyzed for AMP and ATP levels by LC/MS. Data are means ± SE of 3 independent experiments. *P < 0.05, **P < 0.01. n.s., not significant.

The effect of ER stress on AMPK activity was investigated. When MIN6 cells were exposed to tunicamycin, AMPK activity, indicated by levels of p-AMPK and p-ACC, was significantly inhibited (Fig 1A). As mentioned previously, C/EBPβ expression in ordinary conditions is minimal, but was significantly enhanced by ER stress in pancreatic beta cells concomitant with increased levels of p-eIF2α and CHOP (Fig 1A) [5]. We examined the possibility that C/EBPβ, the expression of which is enhanced by ER stress, affects AMPK activity. Overexpression of empty vector in MIN6 cells did not affect the expression of C/EBPβ and ER stress markers (Fig 1B upper left) and the efficiency of transient transfection was dependent on the amount of C/EBPβ DNA used for transfection (Fig 1B, lower left). Overexpression of C/EBPβ in MIN6 cells also caused a significant reduction of AMPK activity, as indicated by the decreased phosphorylation of AMPK and ACC (Fig 1B, middle and right).

We established pancreatic beta cell-specific C/EBPβ TG mice, as previously described [5]. The TG mice did not exceed glucose levels of about 200 mg/dL, indicating that the effect of hyperglycemia remained minimal [16]. Pancreatic islets isolated from TG mice showed significantly decreased AMPK phosphorylation. Taken together, we believe this findings indicate that the reduced AMPK phosphorylation was caused not by hyperglycemia, but rather by accumulation of C/EBPβ (Fig 1C).

To elucidate the link between AMPK activity and C/EBPβ, we evaluated AMP and ATP levels by metabolomic analysis. Compared with wild-type, islets from the TG group had significantly lower AMP levels, but no significant change in ATP levels (Fig 1D). As a result, the AMP/ATP ratio was significantly lowered. Thus, when pancreatic beta cells are exposed to ER stress, C/EBPβ accumulates, lowering the AMP/ATP ratio and AMPK activity.

### C/EBPβ expression levels are dependent on AMPK activity

**Fig 2:**
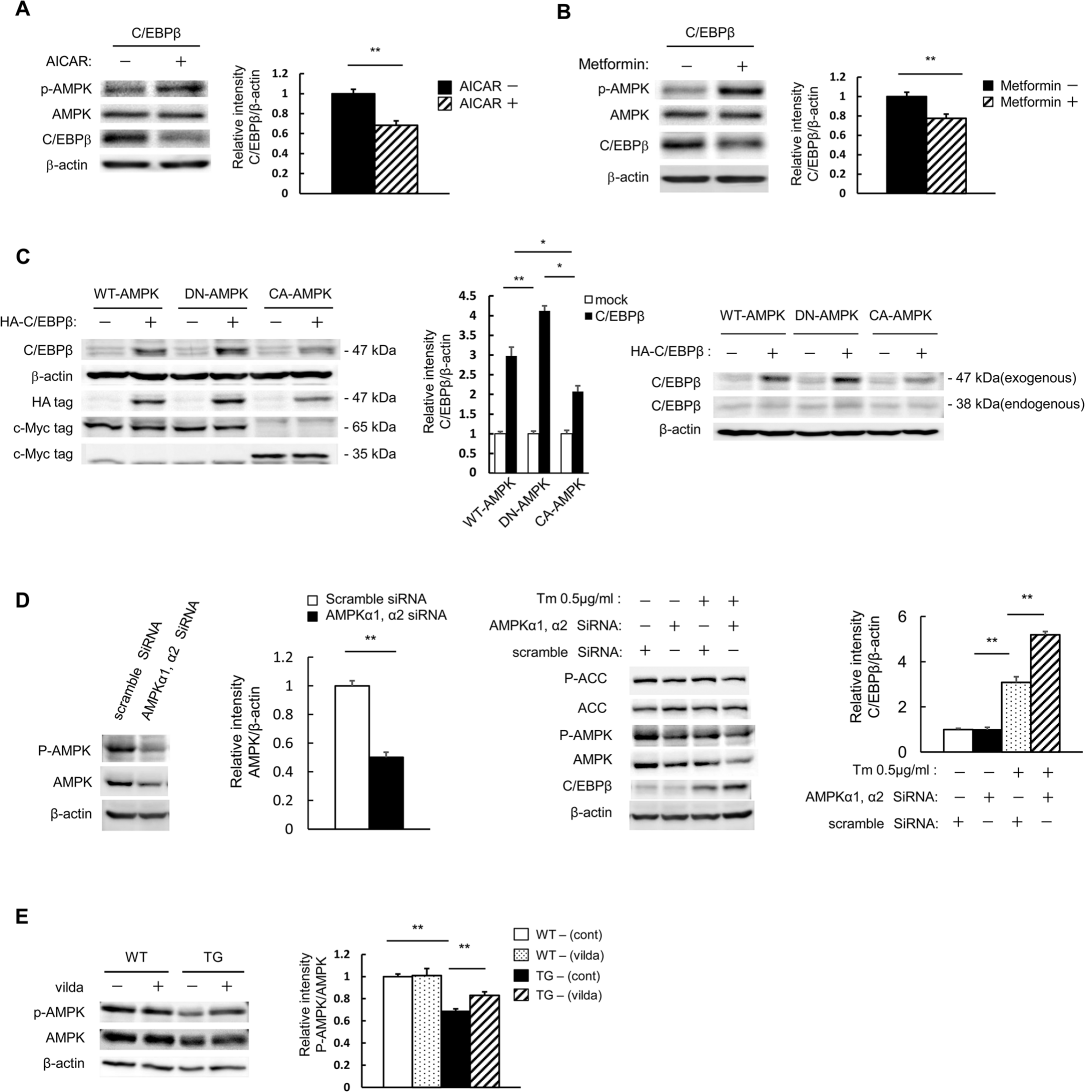
C/EBPβ expression is inhibited when AMPK is activated pharmacologically. A, B: MIN6 cells were transfected with expression vectors for C/EBPβ. The cells were incubated with 100 μM AICAR (A) or 500 μM metformin (B) for 24 h and analyzed with the indicated antibodies (left). Quantitation of C/EBPβ expression is normalized to β-actin (right). C: MIN6 cells were transfected with expression vectors for c-Myc-tagged AMPK, CA-AMPK, DN-AMPK, and/or human influenza hemagglutinin epitope-tagged C/EBPβ, as indicated. These transfected cells were analyzed with the indicated antibody (left and right). Quantitation of C/EBPβ expression is normalized to β-actin (middle). D: MIN6 cells treated with scramble siRNA (control) and AMPK siRNA were lysed and subjected to immunoblot analysis with antibodies against AMPK, ACC, p-AMPK, p-ACC, and C/EBPβ. E: Isolated islets from WT and TG mice following 8 weeks of vildagliptin (vilda) treatment were analyzed with the indicated antibodies (left). Quantitation of the AMPK phosphorylation level is normalized to total AMPK (right). Data are means ± SE of 3 (C, D) or 4 (A, B, E) independent experiments. *P < 0.05, **P < 0.01.

We next examined the effect of AMPK activity on C/EBPβ expression levels in pancreatic beta cells. MIN6 cells were transfected with a C/EBPβ expression vector and subsequently stimulated with 5-amino-1-β-D-ribofuranosyl-imidazole-4-carboxamide (AICAR). The elevated AMPK activity was accompanied by a decrease in C/EBPβ expression (Fig 2A). Similarly, metformin also reduced C/EBPβ expression (Fig 2B). Next, the effect of AMPK on C/EBPβ expression levels was investigated directly with HA-tagged C/EBPβ and c-Myc-tagged C/EBPβ. The exogenous C/EBPβ used here has three HA tags, which was expected to produce a difference of approximately 9 kDa in band mobility (Fig 2C, right). Therefore, judging by the mobility, the band visible in Lanes 1, 3, and 4 in Fig 2C, next to the exogenous C/EBPβ band (47 kDa) in Lanes 2, 4, and 6, are likely to be non-specific and not a band for endogenous C/EBPβ (38 kDa). When C/EBPβ was co-expressed with DN-AMPK, C/EBPβ levels were significantly increased compared with wild-type AMPK co-expression. Moreover, C/EBPβ co-expression with CA-AMPK resulted in significantly reduced C/EBPβ levels (Fig 2C, left and middle). We examined the endogenous C/EBPβ band, and found that normal endogenous C/EBPβ expression levels were low and that no changes were observed even during the coexpression of WT-AMPK; however, when DN-AMPK was coexpressed a slight increase in C/EBPβ expression was noted. Conversely, when CA-AMPK was coexpressed, a slight decrease was observed (Fig 2C, right). To further confirm the effect of AMPK on the expression of C/EBPβ, we established AMPK knockdown MIN6 cells, where AMPK was knocked down with siRNA. AMPK protein was significantly reduced by approximately 50% at the protein level, and phosphorylation of AMPK and ACC was subsequently reduced (Fig 2D, leftmost and second left). As expected, a reduction of AMPK expression created an increase in C/EBPβ expression (Fig 2D, rightmost and second right). Isolated islets were then used to analyze the *in vivo* effects of vildagliptin on TG mice. AMPK phosphorylation, which was decreased in the pancreatic islets of TG mice (Fig 1C and 1D), was significantly increased by vildagliptin (Fig 2E). This enhanced AMPK phosphorylation was thought to be a key factor in the reduction of C/EBPβ expression.

### Phosphorylation is required for the stabilization of C/EBPβ

**Fig 3:**
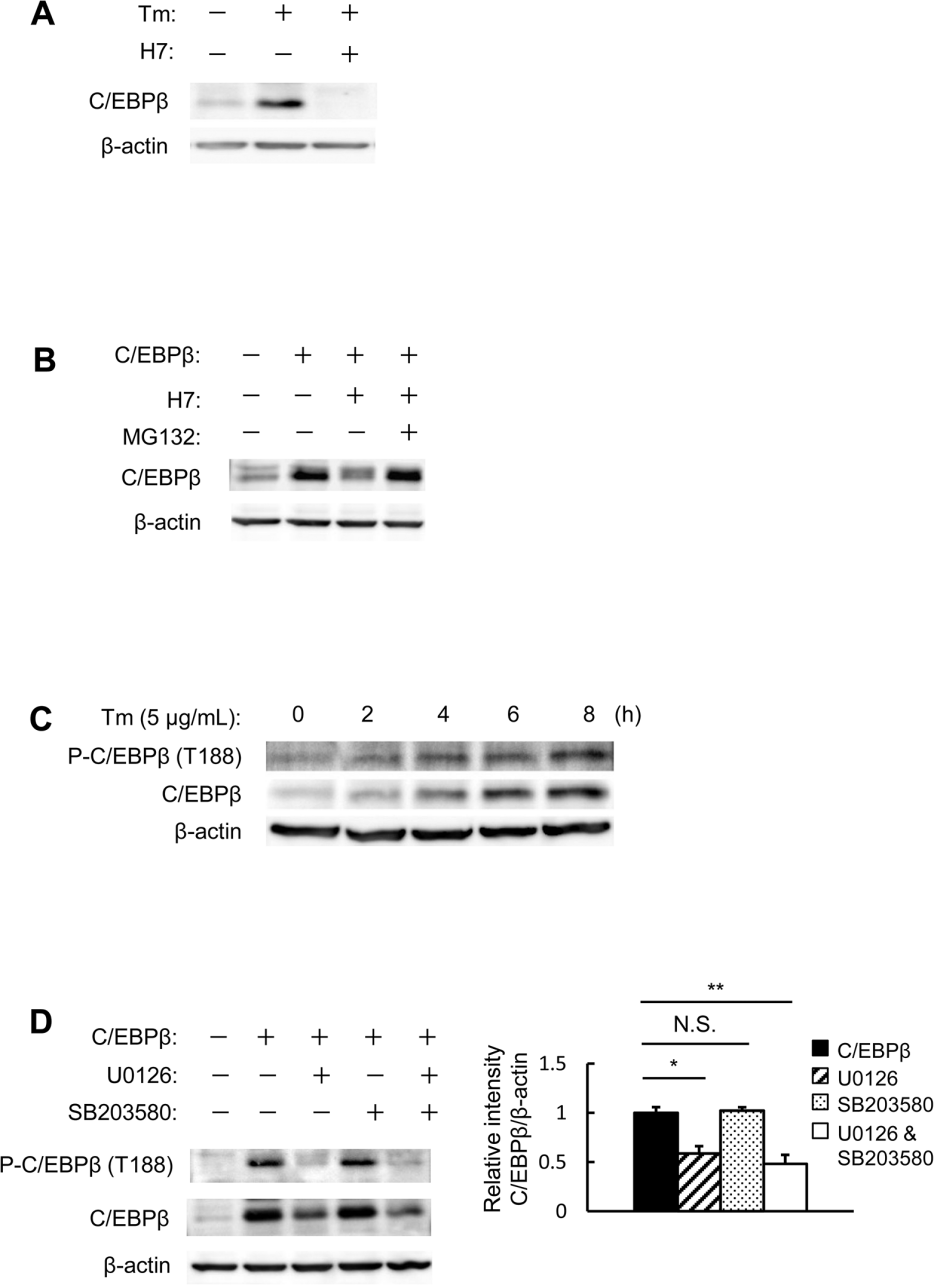
C/EBPβ phosphorylation is required for protein stabilization. A: MIN6 cells were incubated with 2 μg/mL tunicamycin and/or 20 μM H7. B: MIN6 cells were transfected with expression vectors for C/EBPβ and then incubated with 20 μM H7 and 20 μM MG132. C: MIN6 cells were incubated with 5 μg/mL tunicamycin for the indicated periods. C/EBPβ expression and the phosphorylation of C/EBPβ at Thr-188 were analyzed by western blot analysis. D: MIN6 cells were transfected with expression vectors for C/EBPβ and then incubated with 20 μM U0126 and 20 μM SB203580 (left). Quantitation of C/EBPβ expression is normalized to β-actin (right) (n = 3). *P < 0.05, **P < 0.01. n.s., not significant.

Regarding the mechanism by which AMPK regulates C/EBPβ levels, we hypothesized that AMPK might modulate the phosphorylation status of C/EBPβ. The effect of 1-(5-isoquinoline-sulfonyl)-2-methylpiperazine (H7), which is a serine/threonine kinase inhibitor, on C/EBPβ-overexpressing MIN6 cells was therefore investigated. Endogenous C/EBPβ levels were increased after tunicamycin treatment, but C/EBPβ induction was abolished in the presence of H7 (Fig 3A). Next, the effects of H7 on protein stability in C/EBPβ-overexpressing MIN6 cells were investigated. H7 also significantly reduced the levels of overexpressed C/EBPβ (Fig 3B). The impact of inhibiting C/EBPβ expression was almost entirely restored with a proteasome inhibitor, Cbz-Leu-Leu-leucinal (MG132) (Fig 3B). Thus, when the serine/threonine residues of C/EBPβ are phosphorylated, proteasome-mediated degradation of C/EBPβ is inhibited, leading to its accumulation. We then examined the relationship between C/EBPβ levels and its phosphorylation status. Increased C/EBPβ levels in MIN6 cells after tunicamycin treatment were found to be accompanied by C/EBPβ T188 phosphorylation (Fig 3C). Extracellular signal-regulated kinase (ERK) and p38 are known kinases of C/EBPβ T188 [17,18]; therefore, the impact of ERK and p38 on C/EBPβ overexpression was investigated. C/EBPβ levels were decreased by an ERK inhibitor (U0126), but not by a p38 inhibitor (SB203580) (Fig 3D). This suggested that serine/threonine kinases play an important role in the protein stability of C/EBPβ, a role that is played in part through ERK.

### AMPK destabilizes C/EBPβ via T188 dephosphorylation

**Fig 4:**
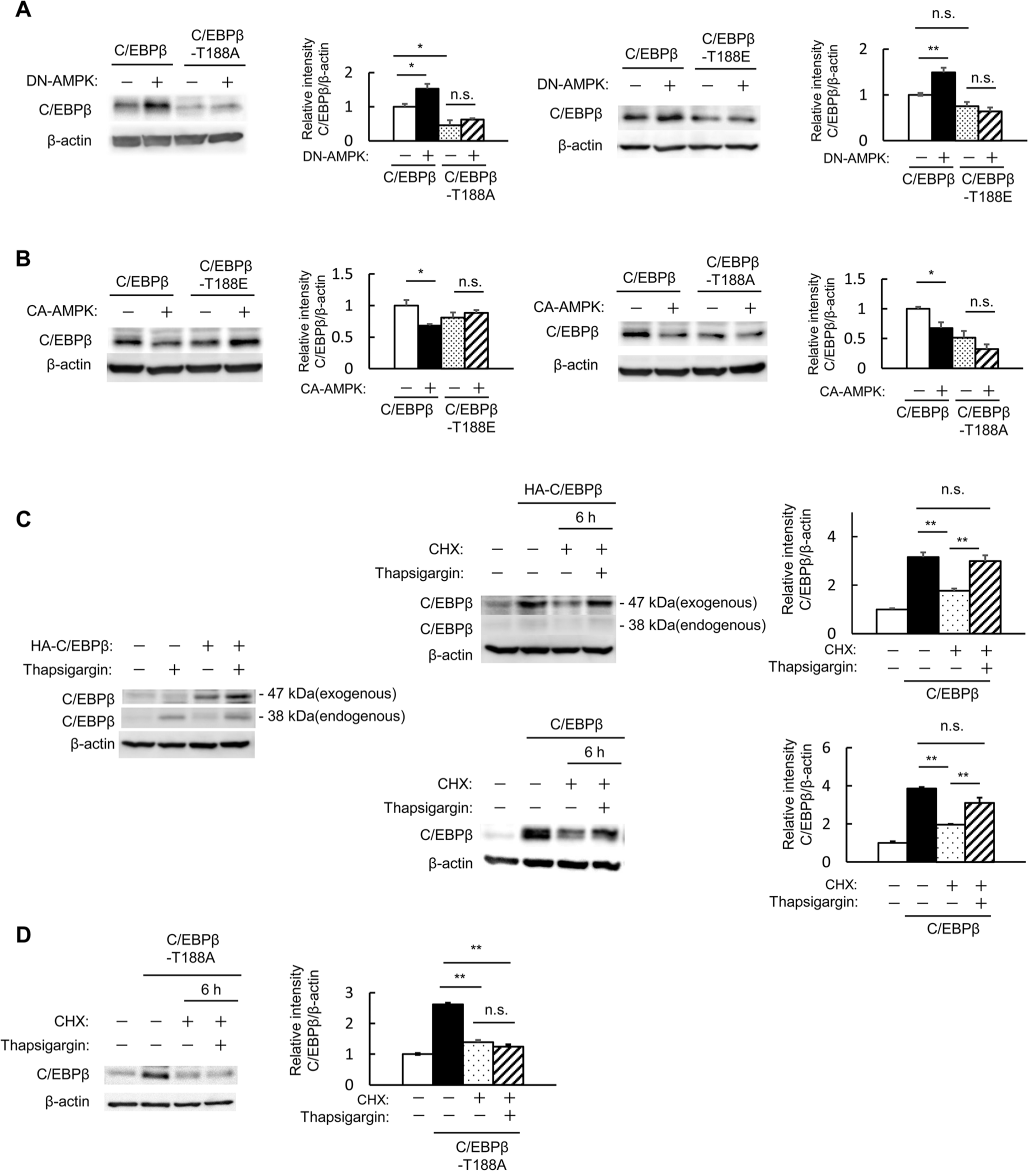
AMPK destabilizes C/EBPβ via T188 dephosphorylation. A: MIN6 cells were co-transfected with expression vectors for C/EBPβ, T188A-mutant C/EBPβ, or T188E-mutant and mock or DN-AMPK (leftmost and second right). Quantitation of C/EBPβ expression is normalized to β-actin (second left and rightmost). B: MIN6 cells were co-transfected with expression vectors for C/EBPβ or T188A-mutant C/EBPβ, T188E-mutant and mock or CA-AMPK (leftmost and second right). Quantitation of C/EBPβ expression is normalized to β-actin (second left and rightmost). C, D: MIN6 cells were transfected with expression vectors for C/EBPβ or HA-tagged C/EBPβ. The cells were pretreated with 1 μg/mL CHX and incubated with 1 μg/mL CHX and 1 μM thapsigargin. C/EBPβ (C) or T188A-mutant C/EBPβ (D) expression was measured by western blot analysis (C; left and middle, D; left). Quantitation of C/EBPβ expression is normalized to β-actin (right). Data are means ± SE of 3 independent experiments. *P < 0.05, **P < 0.01. n.s., not significant.

A non-phosphorylatable mutant (T188A) was generated to investigate the role of T188 phosphorylation in C/EBPβ stability. When C/EBPβ and DN-AMPK were co-expressed, C/EBPβ expression levels were further increased (Figs 2C and 4A). However, this effect was not observed when T188A or T188E mutant C/EBPβ was co-expressed with DN-AMPK (Fig 4A). When wild-type C/EBPβ and CA-AMPK were co-expressed, C/EBPβ levels were reduced, but this was not the case with the T188A or T188E mutant C/EBPβ (Fig 4B). This suggested that AMPK activation leads to the dephosphorylation of C/EBPβ T188, thereby destabilizing C/EBPβ, and that inactivation of AMPK increased C/EBPβ levels via the phosphorylation of T188.

AMPK activity was decreased under ER stress (Fig 1A). In addition to the known increased transcription of C/EBPβ under ER stress, we examined the effects of ER stress on C/EBPβ stability. We evaluated the effect of the ER-stress inducer thapsigargin separately for endogenous (C/EBPβ) and exogenous (HA-C/EBPβ) expression levels. Both were increased by thapsigargin treatment, but that this effect on the endogenous C/EBPβ expression was weaker than that on the HA-C/EBPβ (Fig 4C, left). To examine the possibility that this increase in exogenous C/EBPβ expression is due to improvements in protein stability, we assessed C/EBPβ expression levels after cycloheximide treatment. Cycloheximide treatment of C/EBPβ-overexpressing cells with or without HA-tag reduced C/EBPβ protein levels, but not in cells that were co-stimulated with thapsigargin (Fig 4C, middle and right). In contrast, endogenous C/EBPβ expression did not increase even when stimulation with thapsigargin was performed after cycloheximide treatment in the presence of HA-C/EBPβ (Fig 4C, upper-middle). This seems to be due to the inhibition of endogenous protein synthesis associated with cycloheximide treatment.

However, no stabilizing action of ER stress on C/EBPβ was observed with T188A (Fig 4D). This suggested that AMPK also plays a role in the increased stability of C/EBPβ under ER stress.

### Metformin has no effect on glucose tolerance in pancreatic beta cell-specific C/EBPβ TG mice

**Fig 5:**
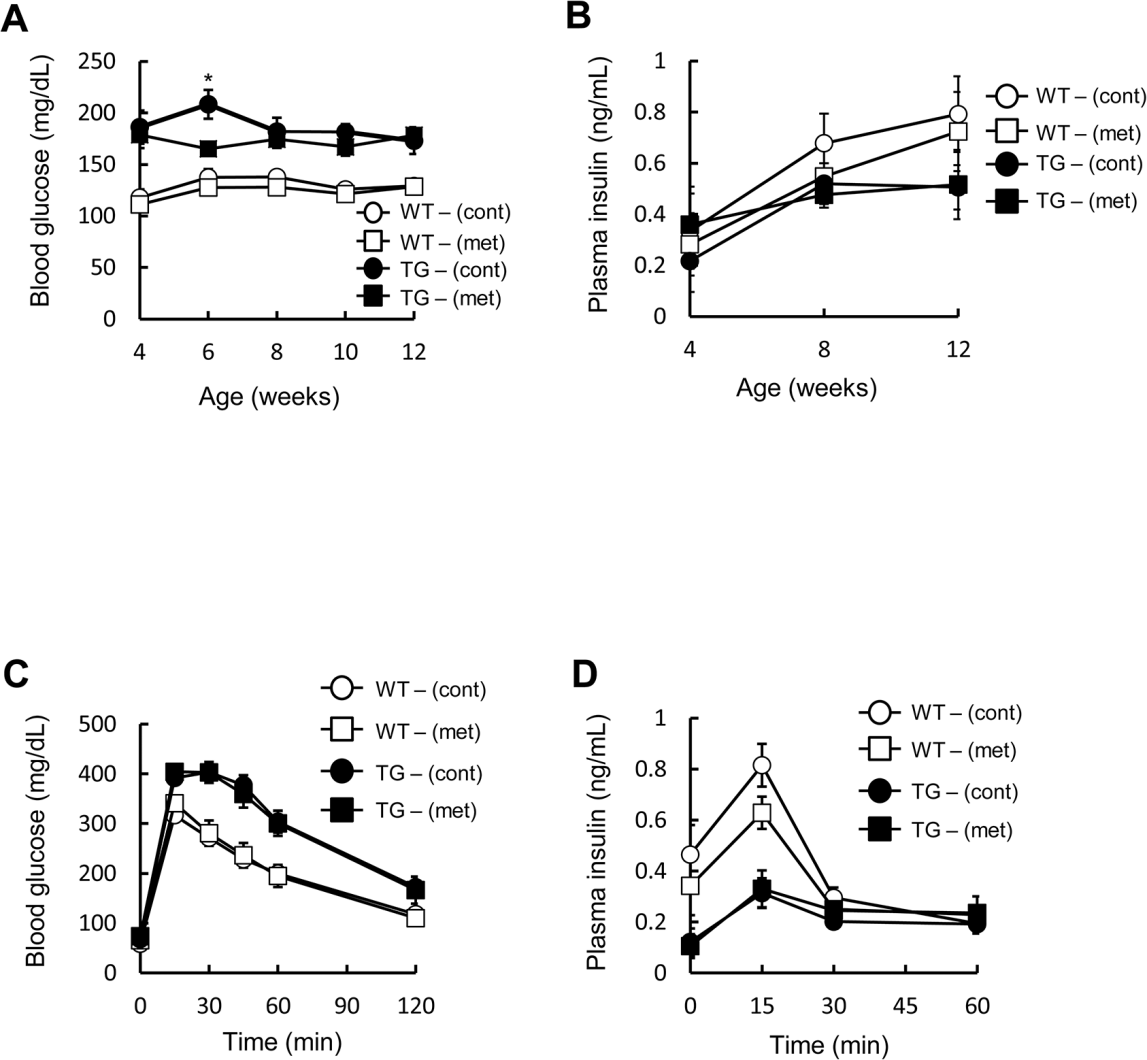
Effect of metformin on glucose tolerance in pancreatic beta cell-specific C/EBPβ TG mice. A: Blood glucose levels and B: plasma insulin concentrations during the 8-week treatment period (n = 10–16 per group). C: Blood glucose levels and D: plasma insulin levels after an oral glucose load (1.5 mg/g body weight) following 8 weeks of metformin (met) treatment (n = 10–13 per group). *P < 0.05.

C/EBPβ accumulation in pancreatic beta cells subjected to ER stress is one of the factors leading to pancreatic beta cell failure and the onset of diabetes [5]. Under such circumstances, we hypothesized that AMPK activity would also be decreased, and that a potential enhancement of AMPK activity *in vivo* might help to reduce C/EBPβ levels. We therefore investigated the effects of metformin, an AMPK agonist, on C/EBPβ expression levels and pancreatic beta cell mass in TG mice. The treatment had no significant impact on the food intake or body weight of either wild-type or TG mice (data not shown). As reported previously [5,16], TG mice exhibited mild hyperglycemia (Fig 5A), and insulin levels were also lowered in comparison to the wild-type animals (Fig 5B). Metformin administration significantly lowered blood glucose levels 2 weeks after treatment compared with the control group, although not significantly thereafter (Fig 5A). Fed insulin levels were unaffected by metformin (Fig 5B). An oral glucose tolerance test revealed impaired glucose tolerance and reduced plasma insulin levels in TG mice as compared with the wild-type animals. However, no significant difference was observed between wild-type and TG mice after metformin treatment (Fig 5C and 5D). This suggested that metformin has no effect on glucose tolerance in the TG mice.

### Metformin has no effect on AMPK activity and a slight effect on C/EBPβ expression levels in pancreatic beta cell-specific C/EBPβ TG mice

**Fig 6:**
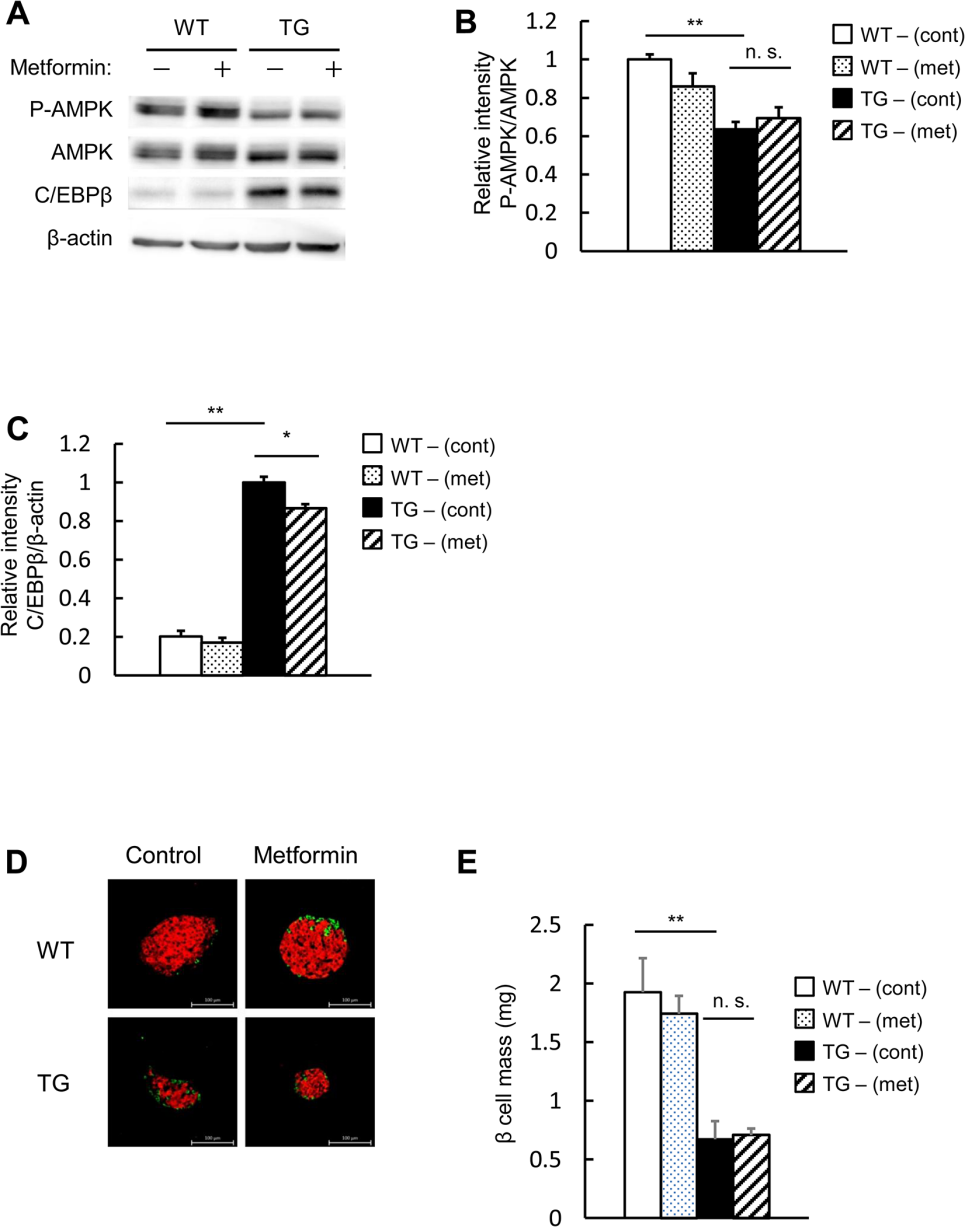
Metformin has no significant impact on AMPK activity or pancreatic beta cell mass in pancreatic beta cell-specific C/EBPβ TG mice. A: Western blot analysis of islets isolated from TG mice following 8 weeks of metformin (met) treatment for analysis with the indicated antibodies. B: Quantitation of the AMPK phosphorylation level is normalized to total AMPK in A (n = 4 per group). C: Quantitation of C/EBPβ expression in A (n = 3 per group). D: Immunofluorescence staining of pancreas sections for insulin (red) and glucagon (green) following 8 weeks of metformin administration. Magnification bar = 100 μm. E: Quantification of beta cell mass in WT and TG mice (n = 5–7 per group). *P < 0.05, **P < 0.01. n.s., not significant.

AMPK activity was assessed using isolated islets. In the islets of TG mice, AMPK activity was reduced in comparison to the wild-type animals (Fig 6A and 6B). Administration of metformin had no impact on AMPK activity in wild-type mice as well as TG mice (Fig 6A and 6B). C/EBPβ, which showed enhanced expression in the islets of the TG mice, was slightly reduced by metformin administration (Fig 6A and 6C). Pancreatic beta cell mass, which was reduced in the TG mice, was largely unaffected by metformin administration (Fig 6D and 6E). This suggested that when metformin is administered to TG mice, there is no recovery of AMPK activity and a mild reduction of C/EBPβ expression levels. However, we were unable to identify a significant impact on pancreatic beta cell mass.

### Combined use of metformin and vildagliptin ameliorates glucose tolerance in pancreatic beta cell-specific C/EBPβ TG mice

**Fig 7:**
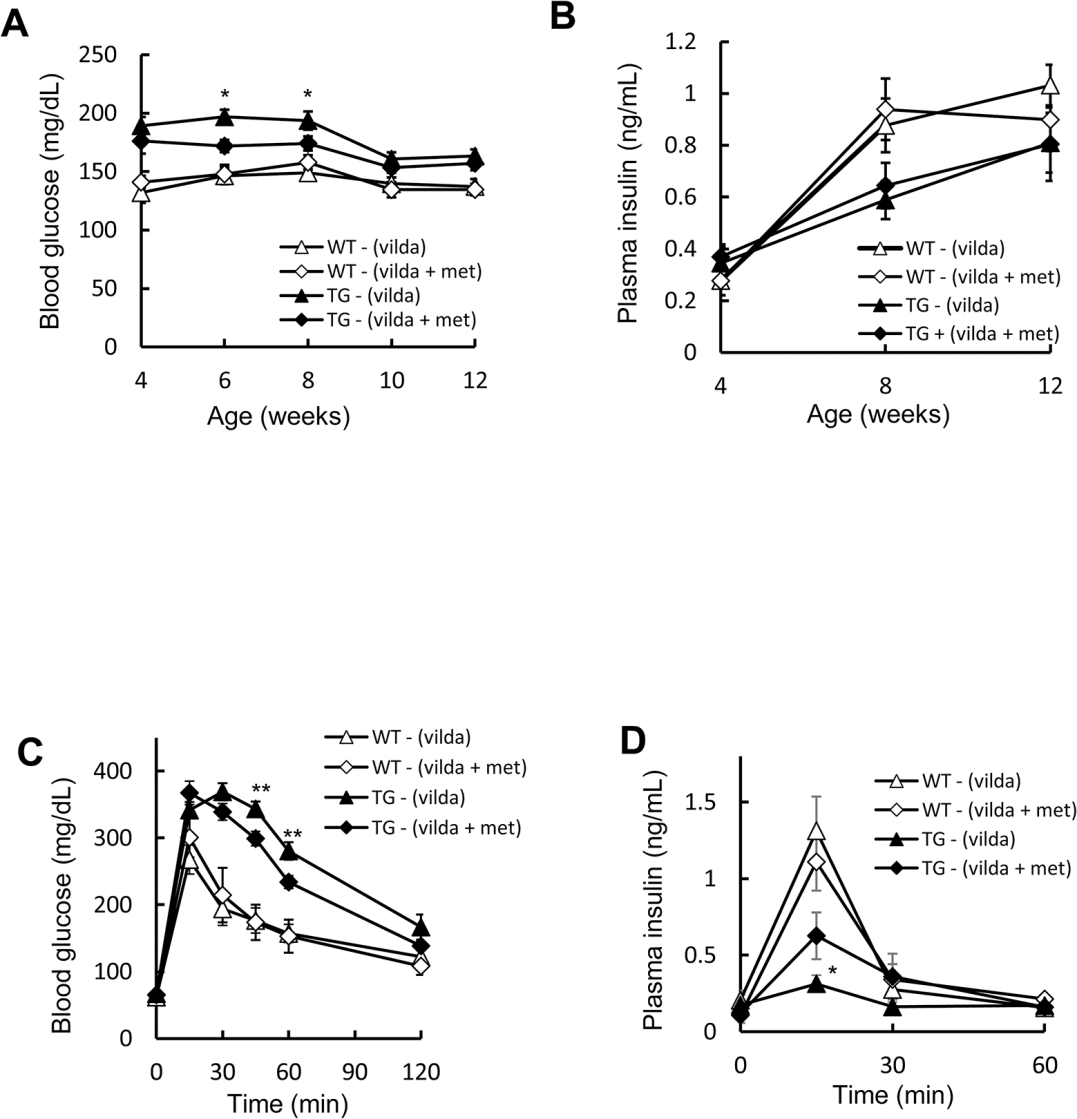
Effect of the combined use of metformin and vildagliptin on glucose tolerance in pancreatic beta cell-specific C/EBPβ TG mice. A: Blood glucose levels and B: plasma insulin concentrations during the 8-week treatment period (n = 11–15 per group). C: Blood glucose levels and D: plasma insulin levels after an oral glucose load (1.5 mg/g body weight) following 8 weeks of vildagliptin (vilda) and/or metformin (met) treatment (n = 5–11 per group). WT-(vilda), open triangles; WT-(vilda+met), open diamonds; TG-(vilda), black triangles; and TG-(vilda+met), black diamonds. *P < 0.05, **P < 0.01.

Previously, we showed that C/EBPβ expression in the islets is reduced and pancreatic beta cell mass is increased when vildagliptin is administered to TG mice concomitantly with amelioration of ER stress and restoration of insulin signaling [16]. Moreover, vildagliptin administration to TG mice recovers islet AMPK phosphorylation (Fig 2D). We therefore tested the combination of metformin with vildagliptin for further enhancement of AMPK phosphorylation. Accordingly, both wild-type and TG groups were administered vildagliptin, and these groups were divided into four groups: those who were administered metformin and those who were not. The combined use of metformin and vildagliptin did not significantly affect body weight (data not shown). Compared with vildagliptin alone, the fed blood glucose levels of the TG mice were significantly reduced in the combined drug group up to and including 4 weeks of administration; however, no difference was observed after 6 weeks of treatment (Fig 7A). Insulin levels showed no significant difference between the vildagliptin alone and combined drug groups (Fig 7B). An oral glucose tolerance test showed a significant improvement in glucose tolerance and insulin secretion in the combined drug group compared with the vildagliptin alone group (Fig 7C and 7D).

### Combined use of metformin and vildagliptin increases pancreatic beta cell mass in pancreatic beta cell-specific C/EBPβ TG mice

**Fig 8:**
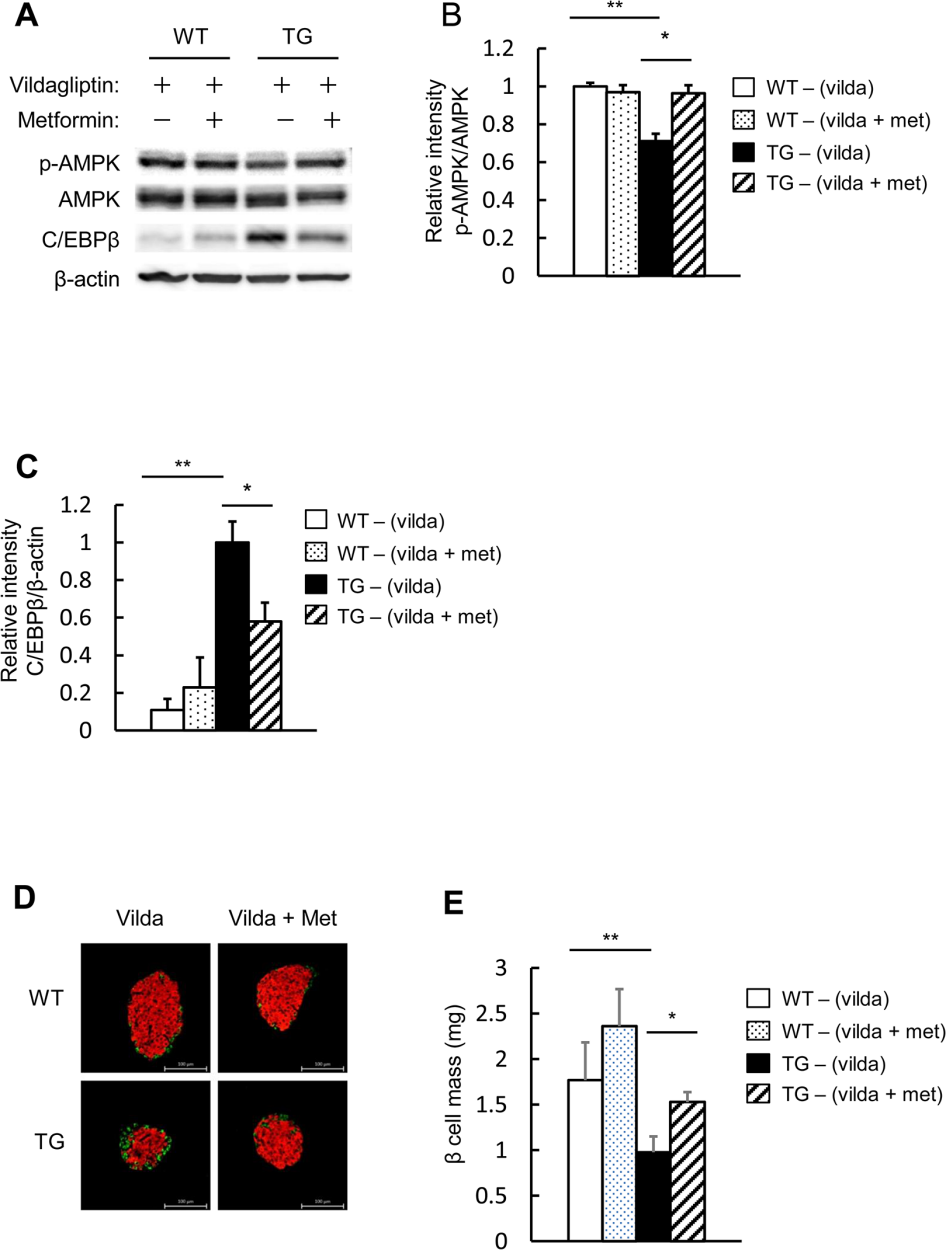
Effect of combined use of metformin and vildagliptin on the islets in pancreatic beta cell-specific C/EBPβ TG mice. A: Western blot analysis of islets isolated from TG mice following 8 weeks of vildagliptin (vilda) and/or metformin (met) treatment for analysis with the indicated antibodies. B: Quantitation of the AMPK phosphorylation level is normalized to total AMPK in A (n = 4 per group). C: Quantitation of C/EBPβ expression in A (n = 4 per group). D: Immunofluorescence staining of pancreas sections for insulin (red) and glucagon (green) following 8 weeks of vildagliptin and/or metformin administration. Magnification bar = 100 μm. E: Quantification of beta cell mass in WT and TG mice (n = 6–7 per group). *P < 0.05, **P < 0.01.

AMPK activity was assessed using isolated islets. When vildagliptin was administered to the TG mice, AMPK activity was elevated compared with the TG mice without vildagliptin (Fig 2D), but in combination with metformin, AMPK activity was elevated even more than with vildagliptin alone (Fig 8A and 8B). In addition, in the TG mice, the combined-metformin group had even further reduced C/EBPβ expression levels than with vildagliptin alone (Fig 8A and 8C), although vildagliptin treatment decreased C/EBPβ expression [16]. The combined-metformin group also had a further increase of pancreatic beta cell mass compared with vildagliptin treatment alone (Fig 8D and 8E), although vildagliptin treatment increased beta cell mass [16]. In summary, compared with vildagliptin monotherapy, its combination with metformin further increased AMPK activity and reduced C/EBPβ levels in the islets of the TG mice, thus further increasing pancreatic beta cell mass.

## Discussion

The present study shows that when C/EBPβ expression is enhanced by ER stress, AMPK activity is decreased due to the lower AMP/ATP ratio. Moreover, this decreased AMPK activity results in the accumulation of C/EBPβ by enhancement of protein stability. Decreased AMPK activity and enhanced C/EBPβ expression might form a vicious cycle that leads to pancreatic beta cell failure.

AMPK is considered an evolutionarily conserved sensor of cellular energy status [19–21]. AMPK is activated by pathological stresses such as low glucose [22], hypoxia [23], ischemia [24], and oxidative stress [25]. Metformin exerts its pharmacological action via AMPK activation [26]. In pancreatic beta cells, the effect of AMPK on pancreatic beta cell mass is unclear and remains controversial [27–30]. However, pancreatic beta cell function impaired by glucose toxicity can reportedly be restored by reducing ER stress by activating AMPK [11].

Opinion is divided regarding whether AMPK activation is reduced [31] or elevated [32] in cells in such a damaged condition. Considering the fact that AMPK acts protectively on cells when activated, AMPK activity is likely reduced or its activation inadequate. We therefore exposed MIN6 cells to ER stress to examine changes in AMPK activity, showing that AMPK activity was reduced. As mentioned above, when pathological stresses activate AMPK for a brief period, the decreased ATP and elevated AMP levels are regarded as being an important factor [33], but it is thought that the accumulation of C/EBPβ following chronic ER stress creates a situation in which AMPK activity is inhibited.

We then investigated whether the modification of phosphorylation affects C/EBPβ stability. In particular, Thr188 was reportedly the most important residue with regard to the stability of C/EBPβ in adipocytes [34].

In our investigation, MIN6 cells subjected to ER stress increased C/EBPβ expression, which was accompanied by T188 phosphorylation. T188 phosphorylation was shown to play an important role in the stabilization of C/EBPβ by ER stress or AMPK inhibition. AMPK reportedly inhibits ERK activity when activated [35], indicating that AMPK could inhibit T188 phosphorylation via inhibition of ERK expression.

The accumulation of C/EBPβ brought about by ER stress forms a vicious cycle with the lowering of AMPK activity, resulting in the reduction of pancreatic beta cell mass. We showed that administering vildagliptin restored AMPK activity in TG mice. GLP-1 signaling reportedly reduces NASH levels in the liver by enhancing AMPK activity [36,37]. This action is thought to be cAMP-independent [38], and the impact of GLP-1 signaling on AMPK activity needs to be examined. Our investigation also suggests that GLP-1 signaling might have a role in restoring AMPK activity in pancreatic beta cells. Future investigations will have to address whether this action is direct or a consequence of ER stress mitigation [39].

We also showed that the oral administration of metformin produced no significant improvement in AMPK activity in the islets of TG mice. However, in combination with vildagliptin, AMPK activation was significantly higher than with vildagliptin alone and brought about a greater inhibition of C/EBPβ expression, along with an increase in pancreatic beta cell mass. These findings demonstrate *in vivo* that the drugs enhance pancreatic beta cell survival by breaking the vicious cycles created by ER stress, C/EBPβ accumulation, and reduced AMPK activity.

In summary, as type 2 diabetes progresses, pancreatic beta cells display decreased AMPK activity along with enhanced C/EBPβ expression, and finally beta cell failure and the onset of diabetes. Part of the protective action of existing anti-diabetes drugs on pancreatic beta cells may therefore be brought about by interrupting the vicious cycle of ER stress, C/EBPβ accumulation, and reduced AMPK activation. T188 phosphorylation of C/EBPβ may well be one of the factors involved in this vicious cycle. A better understanding of the interactions between these factors is essential to clarifying the molecular mechanisms involved in pancreatic beta cell failure. Reduction of C/EBPβ levels in pancreatic beta cells is regarded as perhaps being capable of becoming a novel target of treatment that could result in the protection of pancreatic beta cells from ER stress.
